# Synergistic inhibition of autophagy and neddylation pathways as a novel therapeutic approach for targeting liver cancer

**DOI:** 10.18632/oncotarget.3282

**Published:** 2015-03-13

**Authors:** Ping Chen, Tao Hu, Yupei Liang, Yanan Jiang, Yongfu Pan, Chunjie Li, Ping Zhang, Dongping Wei, Pei Li, Lak Shin Jeong, Yiwei Chu, Hui Qi, Meng Yang, Robert M. Hoffman, Ziming Dong, Lijun Jia

**Affiliations:** ^1^ Cancer Institute, Fudan University Shanghai Cancer Center, Department of Oncology, Shanghai Medical College, Fudan University, Shanghai, China; ^2^ College of Basic Medical Sciences, Zhengzhou University, Zhengzhou, China; ^3^ Department of Immunology, Shanghai Medical College, Fudan University, Shanghai, China; ^4^ Collaborative Innovation Center of Henan Province for Cancer Chemoprevention, Zhengzhou, China; ^5^ College of Pharmacy, Seoul National University, Seoul, Korea; ^6^ AntiCancer Biotech Beijing Co. Ltd., Beijing, China; ^7^ Anticancer, Inc., San Diego CA, USA; ^8^ Department of Surgery, University of California, San Diego, CA, USA

**Keywords:** Neddylation, Autophagy, Apoptosis, MLN4924, Chloroquine

## Abstract

Liver cancer is the second-most frequent cause of cancer death in the world and is highly treatment resistant. We reported previously that inhibition of neddylation pathway with specific NAE inhibitor MLN4924, suppressed the malignant phenotypes of liver cancer. However, during the process, MLN4924 induces pro-survival autophagy as a mechanism of drug resistance. Here, we report that blockage of autophagy with clinically-available autophagy inhibitors (e.g. chloroquine) significantly enhanced the efficacy of MLN4924 on liver cancer cells by triggering apoptosis. Mechanistically, chloroquine enhanced MLN4924-induced up-regulation of pro-apoptotic proteins (e.g. NOXA) and down-regulation of anti-apoptotic proteins. Importantly, the down-regulation of NOXA expression via siRNA silencing substantially attenuated apoptosis of liver cancer cells. Further mechanistic studies revealed that blockage of autophagy augmented MLN4924-induced DNA damage and reactive oxygen species (ROS) generation. The elimination of DNA damage or blockage of ROS production significantly reduced the expression of NOXA, and thereby attenuated apoptosis and reduced growth inhibition of liver cancer cells. Moreover, blockage of autophagy enhanced the efficacy of MLN4924 in an orthotopic model of human liver cancer, with induction of NOXA and apoptosis in tumor tissues. These findings provide important preclinical evidence for clinical investigation of synergistic inhibition of neddylation and autophagy in liver cancer.

## INTRODUCTION

Liver cancer is one of the most common and deadly human malignancies, leading the second cause of cancer-related death worldwide [1]. Currently, chemotherapy is used to treat patients with advanced inoperable liver cancer; however, it is generally ineffective [2–4]. Although several molecular events, such as mTOR, c-Met, MEK, and EGFR/IGF signaling pathways [5, 6], have been shown to be involved in liver carcinogenesis in recent years, limited progress has been made in the targeted therapy of liver cancer [7]. Therefore, further exploration and validation of novel molecular targets of liver cancer are still urgently required.

Post-translational protein neddylation is a process that adds the ubiquitin-like molecule NEDD8 to substrates and thus regulates their conformation, stability, localization and function [8–10]. This process is catalyzed by a cascade comprising the NEDD8-activating enzyme E1 (NAE), NEDD8-conjugating enzyme E2, and substrate-specific NEDD8-E3 ligases [8–10]. The best characterized substrates of neddylation are cullin-family proteins, which are scaffolds of multi-unit Cullin-RING E3 ligase (CRL) complexes [8–10]. NEDD8 conjugation to cullins induces conformational changes and activation of CRL to regulate the turnover of diverse specific CRL substrates with fundamental roles in carcinogenesis and tumor progression. The inactivation of CRL, through inhibiting cullin neddylation, suppresses the growth of cancer cells [11–14]. Up-regulation of NEDD8-catalyzing enzymes (E1/E2/E3) and/or global protein neddylation in human cancer further highlights the important role of neddylation in cancer [15–19].

Targeting the neddylation pathway to inactivate CRL E3 ligases has been shown to be an attractive anticancer strategy, best evidenced by the efficacy of the NAE inhibitor MLN4924 in preclinical studies [11–14]. Due to its potent anti-cancer efficacy and well-tolerated toxicity, MLN4924 has been evaluated in multiple Phase I clinical trials for solid tumors and hematologic malignancies [20–22]. The inactivation of neddylation by MLN4924, either as a single agent, or combined with chemoradiotherapy [23–26], triggers multiple cellular responses involving DNA damage stress [27, 28], cell cycle arrest, apoptosis and/or senescence [25–28] to suppress the growth of cancer cells. However, the protective autophagic response as a pro-survival signal and a mechanism of drug resistance is induced by MLN4924 via modulation of the Deptor-mTOR axis in a broad panel of human cancer cells [20, 29].

In the present report, we hypothesize that inhibition of pro-survival autophagy may augment the efficacy of MLN4924 on liver cancer. As detailed below, we found that autophagy-inhibiting agents sensitized liver cancer cells to MLN4924 both *in vitro* and *in vivo* by inducing NOXA-dependent apoptosis.

## RESULTS

### Autophagy inhibitors enhance MLN4924 efficacy on liver cancer cell proliferation

**Figure 1:**
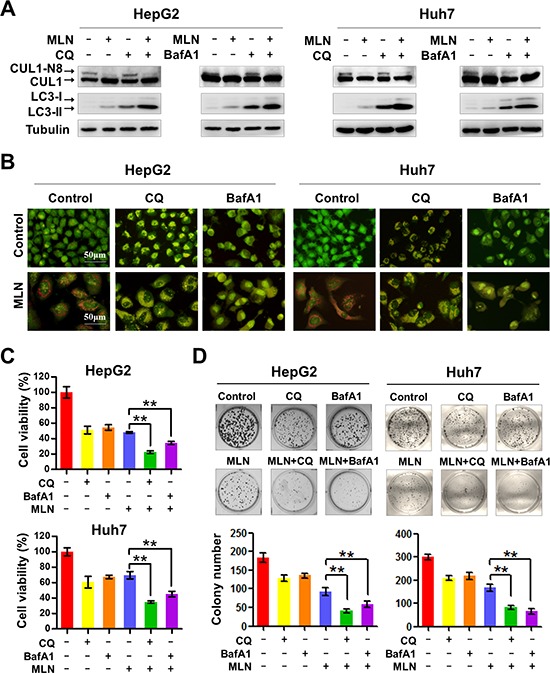
Blockage of autophagy enhances MLN4924-induced suppression of liver-cancer cell proliferation **(A)** Treatment with CQ or BafA1 suppressed cullin neddylation and LC3-II degradation. HepG2 and Huh7 cell lysates were analyzed by immunoblotting with antibodies to cullin1, LC3 and tubulin. Representative images of three independent experiments are presented. **(B)** Treatment with CQ or BafA1 suppressed the formation of AVOs. HepG2 and Huh7 cells were treated with CQ (10 μM), BafA1 (20 nM), with or without MLN4924 (0.33 μM) for 72 hours. Formation of AVOs was examined under fluorescence microscopy. **(C)** Treatment with CQ or BafA1 enhanced MLN4924-induced cell proliferation inhibition. Cell viability was measured using the ATPLite assay (**P < 0.01, *n* = 3). **(D)** The combination of CQ or BafA1 with MLN4924 suppressed colony formation in liver cancer cells. Representative images are shown in the upper panels and statistical results are shown in the lower panels (**P < 0.01; *n* = 3).

Since MLN4924 treatment induces pro-survival autophagy in cancer cells [20, 29], we reasoned that blockage of this protective autophagic response would enhance the effect of MLN4924 on liver cancer growth. To test the hypothesis, two classical autophagy inhibitors CQ and BafA1, which block the late steps of autophagic flux by inhibiting the fusion of autophagosomes with lysosomes and subsequent lysosomal protein degradation [30, 31], were administrated in combination with MLN4924 (MLN4924+CQ or MLN4924+BafA1). As shown in Figure 1A, MLN4924 treatment alone or in combination with CQ or BafA1 specifically inhibited cullin1 (CUL1) neddylation, demonstrating the inactivation of neddylation pathway with these treatments. To determine whether CQ or BafA1 blocks the MLN4924-induced autophagic flux, we first measured the expression of LC3-II, a classical marker of autophagy [30, 31]. Our previous study demonstrated that LC3-II is constantly induced by MLN4924 over time, and it should be further accumulated if its degradation by lysosomes at the late stage of autophagic flux is blocked by CQ and BafA1 [30, 31]. As shown in Figure 1A, the expression of LC3-II was elevated upon MLN4924 treatment due to the induction of the autophagic response and its level was further significantly elevated upon CQ/BafA1 co-treatment with MLN4924 (Figure 1A), indicating that CQ or BafA1 potently blocked the late steps of autophagic flux induced by MLN4924.

Furthermore, using the acridine orange staining assay for autophagy detection, we found that MLN4924 induced intense red acridine orange fluorescence, indicating the formation of acidic vesicular organelles (AVOs), a classical marker of autophagy [30, 31] in treated cells. In contrast, when MLN4924 was combined with either CQ or BafA1, a color shift of acridine orange fluorescence from bright red to a green/dim red was observed, further indicating the inhibition of MLN4924-induced formation of AVOs in cells (Figure 1B).

After establishing the efficacy of MLN4924 on the specific inhibition of cullin neddylation and the efficacy of CQ/BafA1 on the blockage of autophagy signaling, we then determined whether blockage of the autophagic response sensitized liver cancer cells to MLN4924. To test this, cell viability and clonogenic cell survival were evaluated with MLN4924+CQ and MLN4924+BafA1 treatment compared to MLN4924 treatment alone. We found that inhibition of the autophagic response with either CQ or BafA1 significantly enhanced MLN4924-induced inhibition of cell viability (Figure 1C) and clonogenic cell survival (Figure 1D) in both HepG2 and Huh7 cells. These results demonstrated that blockage of the autophagic response significantly enhanced the efficacy of MLN4924 on liver cancer cells (*P* < 0.01).

### Blockage of the autophagy response enhances MLN4924-induced apoptosis

**Figure 2:**
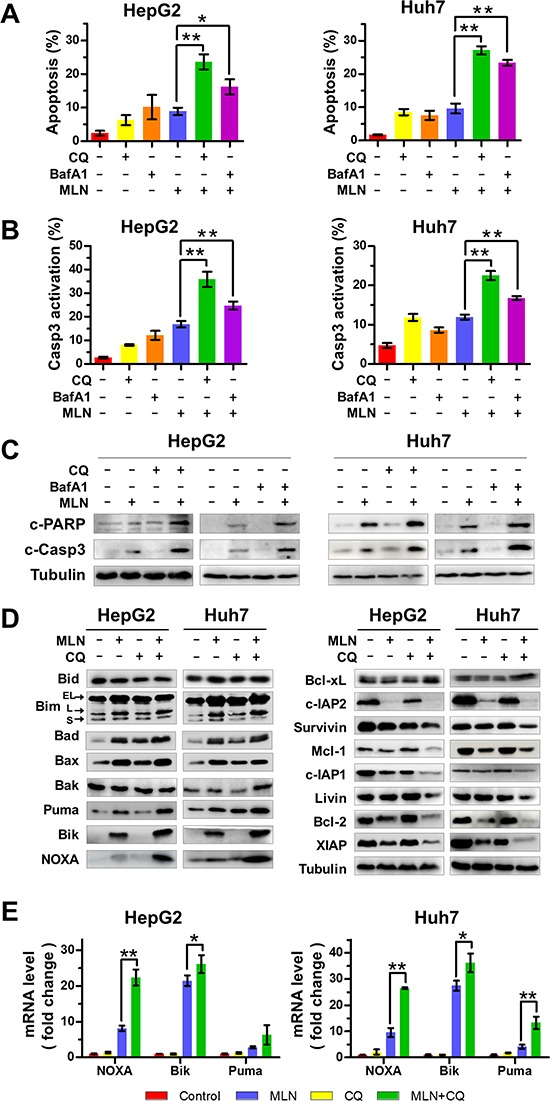
Blockage of the autophagic response increases MLN4924-induced apoptosis of liver-cancer cells **(A-C)** Co-treatment with CQ or BafA1 enhanced MLN4924-induced apoptosis. HepG2 and Huh7 cells were treated with 0.33 μM MLN4924 alone or in combination with CQ (10 μM) or BafA1 (20 nM) for 72 hours. Apoptosis was determined by the Annexin V-FITC/PI double-staining analysis (A). Caspase 3 activity was analyzed by FACS (B) and cleaved PARP and caspase 3 were detected by immunoblotting (C). **(D)** The expression of Bcl-2 family protein was determined by immunoblotting. HepG2 and Huh7 cells were treated with MLN4924 (0.33 μM), CQ (10 μM) or both for 72 hours. Cell extracts were prepared, and equal amounts of protein were separated by SDS-PAGE and subjected to immunoblotting analysis with the indicated antibodies. Tubulin served as a loading control. The immunoblotting data shown here are representative of three independent experiments. **(E)** The mRNA level of *NOXA*, *Bik*, and *Puma* was determined with the Q-PCR assay (*P < 0.05; **P < 0.01, *n* = 3).

We next investigated the underlying mechanisms of enhanced MLN4924 efficacy on liver cancer cells with autophagy blockage. In comparison with MLN4924 alone, MLN4924+CQ or MLN4924+BafA1 treatment significantly increased the Annexin V-positive cell population (Figure 2A), suggesting an amplification of MLN4924-trigered apoptosis in HepG2 and Huh7 cells. Moreover, blockage of autophagy enhanced caspase-3 activity, another indicator of apoptotic induction (Figure 2B). Consistent with the results described above, we found that the expression of cleaved PARP and cleaved caspase 3 were substantially up-regulated upon MLN4924+CQ or MLN4924+BafA1 treatment when compared to MLN4924 alone (Figure 2C).

Besides, previous studies showed that G2 phase cell cycle arrest triggered by MLN4924 also leads to suppression of liver cancer cells [20]. In this study, we found that the elevation of cell cycle arrest triggered by MLN4924+CQ is modest in HepG2 and Huh7 cells compared with MLN4924 treatment alone (Suppl. Figure 1A). Moreover, the expression of p21 and p27, two well-known CRL substrates and cell cycle check-point inhibitors which are accumulated upon treatments, appears to be no significant difference between MLN4924 and MLN4924+CQ (Suppl. Figure 1B). These results indicate that cell cycle arrest is not a main action enhancing MLN4924-induced efficacy after blockage of the autophagy response. Taken together, these findings indicated that blockage of the autophagic response improved the efficacy of MLN4924 mainly by enhancing apoptosis of liver cancer cells.

To further validate the findings described above, the autophagy pathway was genetically inactivated by silencing of ATG7, an essential gene for autophagy induction [32]. As shown in Suppl. Figure 2, down-regulation of ATG7 expression (Suppl. Figure 2A) effectively inhibited MLN4924-induced formation of AVOs in both HepG2 and Huh7 cells (Suppl. Figure 2B), indicating the effective inhibition of the autophagy response by ATG7 knockdown. As a result, silencing of ATG7 significantly increased the Annexin V-positive cell population and the activity of caspase-3 (Suppl. Figure 2C) in MLN4924-treated cells. These results demonstrated that blockage of autophagy via either pharmacological treatment or genetic manipulation enhanced the efficacy of MLN4924 by triggering elevated apoptosis of liver cancer cells.

### Blockage of autophagy induces a coordinated dysregulation of pro-apoptotic and anti-apoptotic Bcl-2 family proteins

To define how autophagy blockage amplified MLN4924-induced apoptosis, CQ, an autophagy inhibitor, was used for the further mechanistic investigation. We first systematically analyzed the expression of a panel of classical pro-apoptotic and anti-apoptotic Bcl-2 family proteins upon treatment of both HepG2 and Huh7 cells, since the balance between pro-apoptotic proteins and anti-apoptotic proteins is crucial for determining the fate of cells. In comparison with MLN4924 alone, MLN4924+CQ treatment induced significant up-regulation of pro-apoptotic proteins including Puma, Bik and NOXA (Figure 2D) and down-regulation of anti-apoptotic proteins including c-IAP1/2, Bcl-2, XIAP and Mcl-1 (Figure 2D) in both cell lines. Quantitative polymerase-chain-reaction (Q-PCR) analysis for the expression of approximately 80 apoptosis-regulatory genes using a human apoptosis PCR array consistently demonstrated that MLN4924+CQ induced an increase in mRNA expression of *NOXA*, *Bik* and *Puma* compared to MLN4924 or CQ alone (Figure 2E and Suppl. Table 3). Among the up-regulated pro-apoptotic proteins, NOXA underwent the most significant increase at both protein and mRNA levels (Figures 2D and 2E), suggesting a more critical role of NOXA in the induction of apoptosis.

### Silencing of NOXA significantly attenuates MLN4924+CQ apoptotic induction and liver-cancer-cell proliferation inhibition

**Figure 3:**
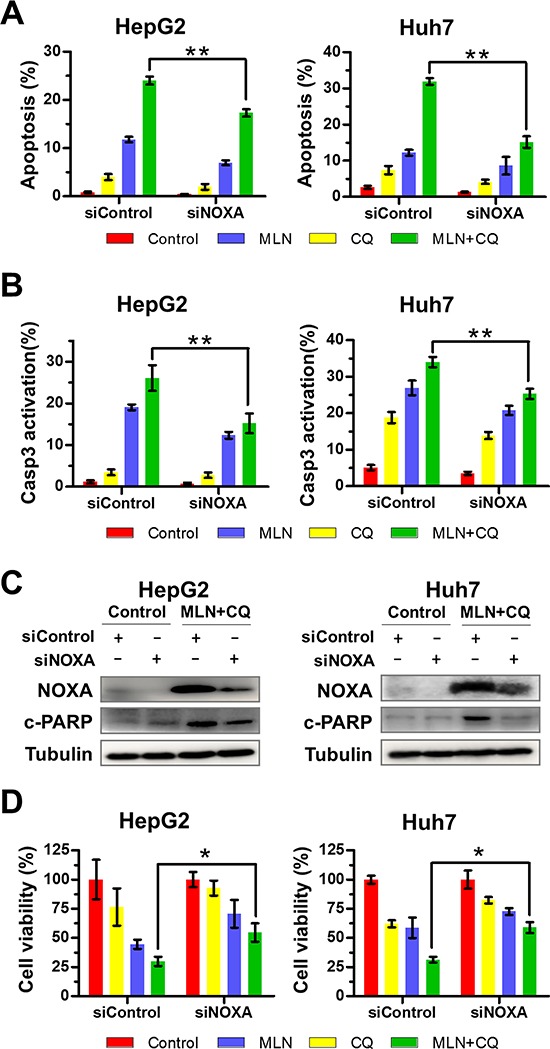
Down-regulation of NOXA significantly attenuates apoptosis and inhibition of liver-cancer-cell proliferation induced by MLN4924+CQ **(A-B)** Down-regulation of NOXA significantly attenuated apoptosis induced by MLN4924+CQ. HepG2 and Huh7 cells were transfected with a control siRNA or NOXA siRNA and were then treated with MLN4924 (0.33 μM), CQ (10 μM), or both for 72 hours. Apoptosis induction was quantified by Annexin V-FITC/PI double-staining analysis (A) or caspase 3 activity analysis by FACS (B). **(C)** Knockdown efficiency and the expression of cleaved PARP were assessed by immunoblotting. **(D)** Cell viability was measured using the ATPLite assay (*P < 0.05; **P < 0.01, *n* = 3).

Next we defined the potential role of NOXA in MLN4924+CQ-induced apoptosis by down-regulating NOXA expression via siRNA silencing. As shown in Figure 3, the down-regulation of NOXA significantly reduced Annexin V-positive (Figure 3A) and caspase-3 positive (Figure 3B) cell populations as well as the expression of cleaved PAPR induced by MLN4924+CQ treatment (Figure 3C), indicating a causal role of NOXA expression in MLN4924+CQ triggered apoptosis in liver cancer cells. Moreover, knockdown of NOXA also substantially attenuated the inhibitory effect of MLN4924+CQ on cell viability (Figure 3D). We observed that siNOXA failed to down-regulate NOXA to the basal level due to the potent induction of NOXA by MLN4924+CQ treatment (Figure 3C), which may underestimate the role of NOXA in MLN4924+ CQ-induced apoptosis. In contrast, silencing of Bik or Puma, which also accumulated upon MLN4924+CQ treatment (Figure 2D), failed to abrogate the suppressive effect of MLN4924+CQ on cell viability in liver cancer cells (Suppl. Figure 3), excluding a major role of Bik and Puma expression in MLN4924+CQ-augmented apoptosis and growth suppression. These findings indicated that MLN4924+CQ-induced apoptosis was partially dependent on the induction of NOXA.

### Enhanced DNA damage contributes to MLN4924+CQ-induced NOXA expression and apoptosis of liver cancer cells

**Figure 4:**
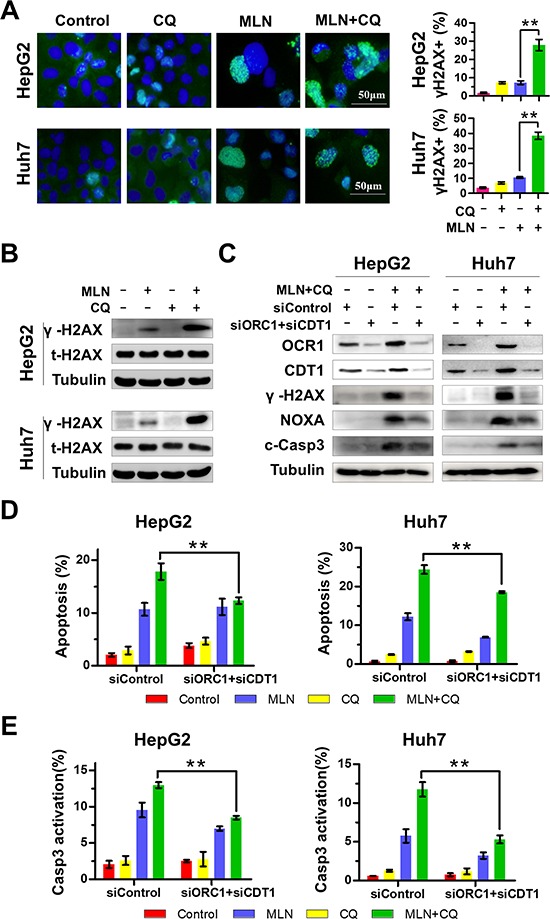
Enhanced DNA Damage contributes to MLN4924+CQ-induced NOXA transactivation and apoptotic induction of liver-cancer cells **(A)** γH2AX foci were determined by immunofluorescence. HepG2 and Huh7 cells were treated with MLN4924 (0.33 μM), CQ (10 μM), or both for 72 hours. γH2AX immunofluorescence was determined and quantified as described in the Materials and Methods. Representative images are shown. **(B)** The expression of total H2AX, γH2AX, ORC1 and CDT1 was determined by immunoblotting. HepG2 and Huh7 cells were treated with MLN4924 (0.33 μM), CQ (10 μM) or both for 72 hours. Cell extracts were prepared, and equal amounts of protein were separated by SDS-PAGE and subjected to immunoblotting analysis with the indicated antibodies. Tubulin served as a loading control. The immunoblotting data shown here are representative of three independent experiments. **(C-E)** Elimination of DNA damage by silencing ORC1 and CDT1 attenuated the expression of NOXA and apoptotic induction by MLN4924+CQ. Cells transiently transfected with control siRNA or the combination of ORC1 and CDT1 siRNA for 24 hours and then were treated with MLN4924 and CQ for 72 hours. Immunoblotting was used to assess knockdown efficiency and the effect on the expression of γH2AX, NOXA and cleaved caspase 3, Tubulin served as a loading control (C). Apoptosis was evaluated by Annexin V-FITC/PI double-staining analysis and caspase 3 activity analysis with FACS (D-E). All data are representative of at least three independent experiments (**P < 0.01, *n* = 3).

NOXA can be induced by DNA damage stress [33, 34] and MLN4924 triggers DNA damage by stabilizing DNA-replication licensing proteins CDT1 and ORC1 as well-identified CRL substrates [27, 28, 35]. Therefore, we proposed that NOXA induction, upon MLN4924+CQ treatment, could be partially attributed to enhanced DNA damage due to CDT1 and ORC1 accumulation. To test this hypothesis, γH2AX, a surrogate marker of DNA double strand breaks, was first analyzed by immunofluorescence staining upon treatment. MLN4924+CQ significantly enhanced the formation of γH2AX foci, compared to MLN4924 or CQ alone, in both HepG2 and Huh7 cells (Figure 4A). The induction of γH2AX was significantly enhanced by MLN4924+CQ when compared to MLN4924 treatment alone as observed by immunoblotting analysis (Figure 4B). To assess the potential role of DNA damage in the induction of NOXA and apoptosis, CDT1 and ORC1 were simultaneously down-regulated by siRNA to block DNA damage upon MLN4924+CQ treatment. As expected, knockdown of CDT1 and ORC1 significantly reduced the expression of γH2AX (Figure 4C), indicating the attenuation of DNA damage by MLN4924+CQ treatment. As a result, the expression of NOXA, cleaved-caspase 3 (Figure 4C), the percentage of Annexin-V-positive (Figure 4D) and caspase-3 active cells (Figure 4E) were significantly reduced when DNA damage stress was abrogated by MLN4924+CQ. These results implied that DNA damage, triggered by CDT1 and ORC1 accumulation, partially contributed to NOXA induction and apoptosis by MLN4924+CQ treatment.

### The production of excessive ROS contributes to MLN4924+CQ-induced NOXA expression and apoptosis in liver cancer cells

**Figure 5:**
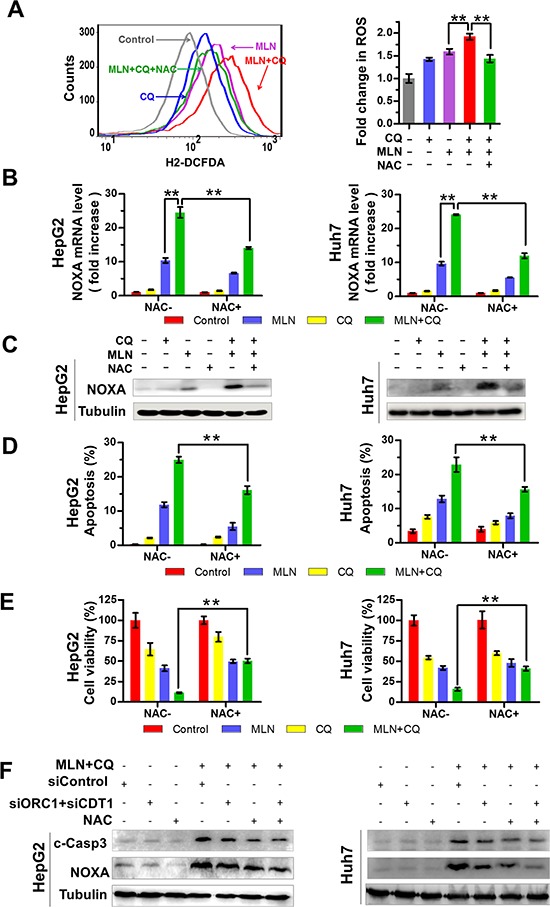
Production of excessive ROS contributes to MLN4924+CQ-induced NOXA transactivation and apoptotic induction of liver-cancer cells **(A)** Effect of MLN4924 or CQ alone or the MLN4924+CQ combination, on ROS generation. HepG2 and Huh7 cells were pre-treated with NAC (50 μM) for 2 hours. Cells with, or without NAC, were treated with MLN4924 (0.33 μM), CQ (20 μM), or both for 4 hours. ROS generation was determined by H2-DCFDA staining and flow cytometry (**P < 0.01, *n* = 3). **(B-C)** Effect of NAC on mRNA (B) and protein (C) expression of NOXA was determined by Q-PCR and immunoblotting respectively (**P < 0.01, *n* = 3). **(D-E)** The antioxidant NAC rescued cell viability and diminished drug-induced apoptosis. HepG2 and Huh7 cells were pre-treated with NAC (50 μM) for 2 hours, and then were treated with MLN4924 (0.33 μM), CQ (20 μM), or both for 36 hours. Apoptotic cells were determined by Annexin V-FITC/PI double-staining through FACS analysis (D). Cell viability was measured using ATPLite assay (E). **(F)** Effect of combined treatment of both NAC and siORC1/CDT1 on NOXA expression and apoptosis induction. The expression of CDT1, ORC1, c-Caspase3 and NOXA was determined by immunoblotting. Tubulin served as a loading control. All data are representative of at least three independent experiments.

NOXA-dependent apoptosis can be partially, but not completely abrogated, by the blockage of DNA damage (Figure 4). To further elucidate the underlying mechanisms of MLN4924+CQ-induced NOXA expression, we investigated the potential role of ROS in NOXA expression, since ROS production during cellular stresses could trigger NOXA expression [36, 37] and MLN4924 induced elevation of ROS generation [20]. We first assessed the impact of drug treatment on mitochondrial transmembrane potential since the impaired mitochondrial electron transport chain serves as a major source of ROS production [26]. As shown in Suppl. Figure 4, treatment of cells with MLN4924+CQ caused significantly enhancement of mitochondrial depolarization when compared to MLN4924 or CQ treatment alone (Suppl. Figure 4). Next, ROS production was monitored with the cell permeable ROS indicator, 2′, 7′-dichlorodihydrofluorescein diacetate (H2-DCFDA), using flow-cytometry analysis [26]. MLN4924 or CQ alone increased the level of ROS while MLN4924+CQ significantly amplified the production of ROS compared to treatment alone (Figure 5A left panel). Moreover, the enhanced production of ROS induced by MLN4924+CQ was completely blocked by pre-treatment of cells with NAC, a classical ROS scavenger [36, 37] (Figure 5A right panel), which significantly down-regulated NOXA expression at both mRNA and protein level (Figure 5B and 5C). And reduction of ROS by NAC also attenuated apoptosis and reduced cell death (Figure 5D and 5E). These results demonstrated that enhanced production of ROS contributed to the induction of NOXA and apoptosis upon MLN4924+CQ treatment in liver cancer cells.

Finally, we determined the effect of combined treatment of both NAC and siORC1/CDT1 on NOXA expression and apoptosis induction. We found that the combination of NAC and siORC1/CDT1 further reduced the expression of NOXA and cleaved-caspase 3 compared to NAC or siORC1/CDT1 alone (Figure 5F). These findings further support the conclusion that both enhanced DNA damage and elevated ROS production contributed to the induction of NOXA and apoptosis.

### Blockage of autophagy enhances the antitumor efficacy of MLN4924 in an orthotopic model of human liver cancer

**Figure 6:**
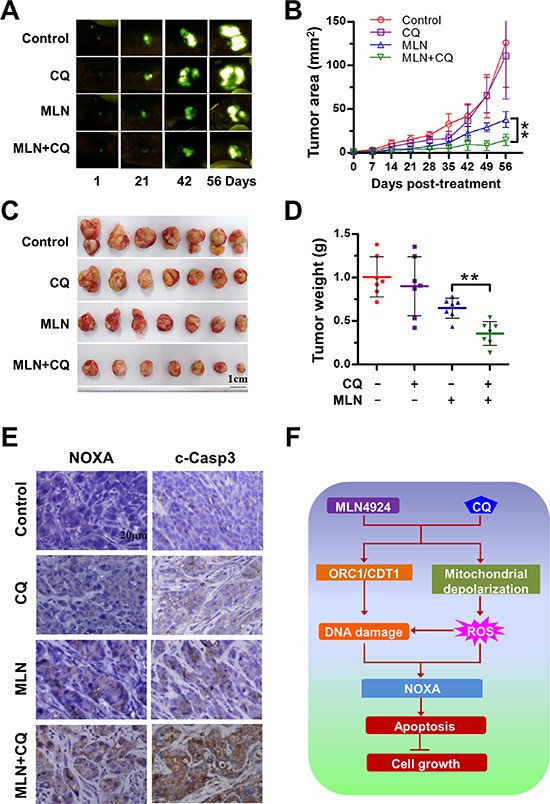
Blockage of autophagy enhances the antitumor efficacy of MLN4924 *in vivo* **(A-B)** MLN4924+CQ significantly inhibited tumor growth compared to MLN4924 or CQ alone measured by fluorescence imaging. Nude mice with HepG2-GFP orthotopic human liver cancer were administrated with MLN4924, CQ, or both according to the Materials and Methods. Tumor size was monitored twice a week by fluorescence imaging (A). The data were converted to tumor growth curves by ModFit LT software (B). **(C-D)** Co-treatment significantly reduced tumor volume compared to MLN4924 or CQ alone. Mice were sacrificed on the 56th day after treatment (the end of study, *n* = 7). Tumor tissues were harvested, photographed (C) and weighed (D) (**P < 0.001). No obvious toxicity against body weight gain was observed during treatment (Suppl. Figure 5). The body weight of animals was measured twice a week during the treatment period. **(E)** Co-treatment enhanced the expression of cleaved caspase 3 and NOXA compared to treatment with MLN4924 or CQ alone *in vivo*. Tumor tissues were collected; paraffin embedded. The expression of cleaved caspase 3 and NOXA was detected using immunochemistry. **(F)** Schema of enhancement of apoptosis of liver cancer cells by combination treatment with MLN4924 and CQ.

We investigated the sensitizing effect of autophagy blockage with CQ on the efficacy of MLN4924 in a clinically-similar orthotopic model of HepG2-GFP liver cancer. Fluorescent HepG2-GFP tumors allowed the determination of tumor growth and progression in real time by external and noninvasive whole-body optical imaging [38, 39]. Compared to MLN4924 treatment alone, MLN4924+CQ inhibited tumor growth (Figure 6A-6C) (*P* < 0.01) and reduced tumor weight (*P* < 0.01) (Figure 6D). During the treatment period, no obvious side effects, such as body weight loss, were observed (Suppl. Figure 5), indicating the combination treatment was well tolerated by the animals. To evaluate the enhanced antitumor mechanism of MLN4924+CQ *in vivo*, tumor tissue sections were analyzed immunohistochemically for the expression of NOXA and cleaved caspase 3, as apoptotic markers. MLN4924+CQ substantially enhanced the expression of NOXA and cleaved caspase 3, indicating the activation of apoptosis in the treated tumors (Figure 6E). These results demonstrated that the inhibition of autophagy pathway sensitized cells to MLN4924 by inducing NOXA-dependent apoptosis *in vivo* as well as *in vitro*.

## DISCUSSION

Liver cancer ranks one of the most common human malignancies worldwide, while the novel strategies for the treatment of this deadly disease are urgently needed [1–4]. Recently, targeting protein neddylation pathway has emerged as an attractive anticancer strategy, as best supported by the discovery of the specific NAE inhibitor MLN4924 in preclinical and clinical trials [8–14]. Moreover, the discovery of overactivation of the neddylation pathway in human cancer further enhances rationality of targeted therapy by inhibiting this pathway [15–19]. However, during the process of targeting this oncogenic pathway for anticancer therapy, pro-survival autophagy was induced as a mechanism of drug resistance in treated cells [20, 29]. In this study, we validated the strategy of blockage of the autophagy response with both pharmaceutical and genetic approaches to sensitize liver cancer cells to investigational neddylation inhibitor MLN4924, both *in vitro* and *in vivo*, due to the induction of exacerbated apoptosis (Figure 6F). These preclinical findings strongly support the further clinical investigation of the combination therapy of autophagy-inhibitory agents with MLN4924 in patients with liver cancer.

Mechanistically, induction of apoptosis by dual inhibition of autophagy and the neddylation pathway is mainly attributed to the induction of proapoptotic BH3-only proteins (Figure 6F). Functional validation demonstrated that the induction of NOXA, but neither Bik nor Puma plays a critical role in the induction of apoptosis upon MLN+CQ treatment. Previous studies reported that MLN4924 either used as a single agent or in combination of chemoradiotherapy also induced the expression of BH3-only proteins to trigger apoptosis in multiple cancer cells [15, 26]. These collective findings highlight a critical role of the up-regulation of BH3-only pro-apoptotic proteins in the induction of apoptosis by targeting neddylation pathway. In addition, we observed that MLN+CQ treatment also induced the down-regulation of several classical anti-apoptotic proteins including c-IAP1/2, Bcl-2, XIAP and Mcl-1 (Figure 2D). It is likely that the down-regulation of these anti-apoptotic proteins cooperates with NOXA to trigger apoptosis.

The induction of NOXA upon dual inhibition of autophagy and the neddylation pathway in our study was partially attributable to enhanced DNA damage stress. Previous studies demonstrated that MLN4924 induces the accumulation of CRL substrates CDT1 and ORC1, two DNA-replication licensing factors, to trigger DNA rereplication stress and induce DNA damage stress [28]. In this study, we found that the combination of CQ with MLN4924 provoked enhanced DNA damage when compared to MLN4924 alone whereas the attenuation of DNA damage stress via simultaneous silencing of CDT1 and ORC1 significantly reduced NOXA expression and apoptotic induction upon MLN+CQ treatment (Figures 4, 6F). These findings demonstrated a casual role of CRL-CDT1/ORC1 axis in the induction of DNA damage stress, NOXA expression as well as apoptotic induction for MLN+CQ-mediated anticancer therapy.

Oxidative stress induced by MLN+CQ also contributes to the induction of NOXA, based on the following findings that (a) MLN+CQ-induced mitochondrial depolarization which impaired mitochondrial electron transport chain as a major source of ROS production; (b) ROS level is significantly elevated upon MLN+CQ treatment; and more importantly (c) the addition of the antioxidant GSH-mimetic NAC, a classical scavenger of ROS, dramatically reduced NOXA expression, attenuated apoptotic induction and cell growth suppression induced by MLN+CQ (Figures 5, 6F). Meanwhile, ROS stress triggered by MLN+CQ may contribute to the induction of exacerbated DNA damage since oxidant stress serves as classical inducer of DNA damage [26] and MLN+CQ induced the production of excessive ROS in treated cells. In support of this hypothesis, two recent investigations showed that MLN4924 either as a single agent [20] or in combination of cisplatin [26] induced elevated ROS generation and oxidative DNA damage to trigger apoptosis.

An important question to be addressed in future studies is the mechanism by which NOXA transactivation is induced by MLN+CQ treatment. Tumor suppressor p53 which is frequently mutated in human cancer [40] is often involved in the transcriptional activation of pro-apoptotic proteins including NOXA [41–43]. However, the induction of NOXA upon MLN+CQ treatment appears p53-independent. We found that NOXA was induced in both wild-type p53-expressing HepG2 cells [44, 45] and mutant/inactivated p53-carrying Huh7 cells [44, 45]. Similarly, previous studies showed that, regardless of the status of p53, NOXA could be transcriptionally activated in response to the treatment of cancer cells with a set of anticancer agents, including pan-proteasome inhibitor botezomib [46, 47]. Future studies will warrant to further address the underlying mechanisms of NOXA transactivation in MLN+CQ-treated cells.

Targeting autophagy pathway with autophagy inhibitors has been developed as an important strategy to sensitize cancer cells to chemotherapeutics that induces autophagic response as a pro-survival signal and a major mechanism of drug resistance [24, 26]. CQ or its derivative hydroxychloroquine (HCQ), the first generation autophagy inhibitors, has been approved by the FDA due to their effectiveness *in vivo* and safety in clinical trials [48], although the underlying mechanisms responsible for the action of CQ/HCQ have not been well addressed. In the present report, we, for the first time, demonstrated the efficacy of autophagy inhibition with CQ on neddylation pathway-targeted therapy for human liver cancer. Our encouraging results obtained from this study provide a strong rationale for the further clinical evaluation of combination with clinically applicable autophagy inhibitors (e.g. CQ and its derivatives) and neddylation inhibitors (e.g. MLN4924) for the treatment of patients with liver cancer. Moreover, these strategies probably have a broader application for other types of human cancers since the induction of pro-survival autophagy by neddylation inhibition is a general phenotype in many human cancer cells [20, 29]. At the molecular level, increased DNA damage stress, excessive ROS production, transactivation of NOXA and induction of apoptosis may serve as biomarkers for determining the therapeutic response of cancer patients to this combination treatment, which is yet to be investigated in future clinical studies.

## MATERIALS AND METHODS

### Cell lines, culture, and reagents

Human liver cancer cell lines HepG2 and Huh7 were obtained from the American Type Culture Collection, and cultured in Dulbecco's Modified Eagle's Medium (Hyclone), containing 10% FBS (Biochrom AG) and 1% penicillin–streptomycin solution, at 37°C with 5% CO_2_. MLN4924 was synthesized and prepared as described [20]. For *in vitro* studies, MLN4924 was dissolved in dimethyl sulfoxide (DMSO) and stored at –20°C. For *in vivo* studies, MLN4924 was dissolved in 10% 2-hydroxypropyl-β-cyclodextrin (HPBCD). The solution of MLN4924 was freshly prepared every week and stored in the dark at room temperature before use.

### Cell viability and clonogenic survival assay

Cells were plated in 96-well plates (3 × 10^3^ cells per well) and treated with drugs as indicated. Cell proliferation was determined using the ATPLite Luminescence Assay kit (PerkinElmer) according to the manufacturer's protocol.

For the clonogenic assay, 500 cells were seeded into 60-mm dishes in triplicate, treated with MLN4924, autophagy inhibitor chloroquine (CQ) or BafilomycinA1 (BafA1) alone, or in combination (MLN4924+CQ or MLN4924+BafA1), followed by incubation for 9 days. The colonies were fixed, stained and counted under an inverted microscope (Olympus, Tokyo, Japan). Colonies with 50 cells or more were counted.

### Acridine orange (ao) staining for autophagy detection

Acridine orange (Sigma, St. Louis, MO) staining was performed to detect autophagy according to a published protocol [30, 31]. Briefly, cells were treated and stained with 1 mM acridine orange in PBS containing 5% FBS at 37°C for 15 minutes. Cells were washed and observed under fluorescence microscopy (magnification: 20 × 100; Olympus BX-51, Olympus Inc., Tokyo, Japan).

### SiRNA knockdown of ATG7, NOXA, Bik, Puma, ORC1 and CDT1

HepG2 and Huh7 cells were transfected with siRNA oligonucleotides, synthesized by RIBOBIO (Guangzhou, China) using Lipofectamine 2000. The siRNA sequences are shown in Suppl. Table 1. Cells transiently transfected with control siRNA or the target gene siRNA were treated with MLN4924, CQ or MLN4924+CQ for 72 h after 24 h transfection.

### Immunoblotting

Cell lysates were prepared for immunoblotting, using antibodies against cullin1 (Santa Cruz, Dallas, Texas), phospho-H2AX at Ser139 (γH2AX), ATG7, cleaved caspase 3, cleaved PARP, Pro-Apoptosis Bcl-2 Family Antibody Sampler Kit, Pro-Survival Bcl-2 Family Antibody Sampler Kit, IAP Family Antibody Sampler Kit, ORC1, CDT1 (Cell Signaling, Boston, MA), tubulin (Likun Trade Co., China), NOXA (Millipore, Billerica, MA) and LC3 (Sigma, St. Louis, MO).

### Apoptosis detection

Cells were treated with the indicated concentration of MLN4924, CQ, BafA1, MLN4924+CQ or MLN 4924+BafA1 for 72 hours. Apoptosis was determined with the Annexin V-FITC/PI Apoptosis Kit (BioVision, Inc. Milpitas, California) and CaspGLOW Fluorescein Active Caspase-3 Staining Kit (BioVision) according to the manufacturer's instructions.

### Evaluation of mitochondrial membrane depolarization

HepG2 and Huh7 cells were treated with drugs as indicated. Mitochondrial Membrane Depolarization was detected with the mitochondrial membrane potential assay kit with JC-1 according to the manufacturer's protocol (Yeasen, Shanghai, China). JC-1 is a potentiometric dye that exhibits a membrane potential-dependent loss as JC-1 aggregates (polarized mitochondria) transition to JC-1 monomers (depolarized mitochondria). The loss of membrane potential is indicated by the fluorescence emission shift from red to green. The levels of fluorescence intensity of the cells were analyzed by flow cytometry (Becton Dickinson FACScan) under an excitation wavelength of 488 nm and emission wavelengths of 530 nm for green fluorescence and 585 nm for red fluorescence.

### Immunofluorescence staining

HepG2 and Huh7 liver cancer cells were plated on chamber slides and treated with MLN4924, CQ or both. Cells were fixed with 4% paraformaldehyde, permeabilized using 0.2% Triton X-100, and incubated with γH2AX primary antibody and Alexa Fluor^®^ 488 Goat Anti-Rabbit IgG (H+L) secondary antibody (Beyotime, Hangzhou, China) respectively. The nuclei were stained by DAPI (Beyotime). Images were captured using fluorescence microscopy (magnification: 20 × 100; Olympus BX-51, Olympus Corp., Tokyo, Japan).

### Quantification of reactive oxygen species

ROS production was monitored with the cell permeable ROS indicator, 2′, 7′-dichlorodihydrofluorescein diacetate (H2-DCFDA) (Sigma), as described by Cossarizza et al [49]. The functional role of ROS generation in apoptosis was assessed using the free-radical scavenger NAC (Beyotime). Cells were pre-incubated with 50 μM NAC for 2 h, followed by co-incubation with indicated chemicals and assessment of apoptosis or ROS generation as described above.

### Rna isolation and quantitative polymerase chain reaction (q-pcr)

Total RNA was extracted using an Ultrapure RNA kit (CWbiotech). RNA (1.0 μg) was purified and reversely transcribed by PrimeScript^®^ RT Master (Takara, Dalian, China) following the manufacturer's instructions. The cDNA was quantified by real-time quantitative PCR using SYBR^®^ Green Real-Time PCR Master Mixes (Applied Biosystems, Foster City, Calif.) and a 7500 Real-time PCR system (Applied Biosystems) according to the manufacturer's instructions. For each sample, the mRNA abundance was normalized to the amount of GAPDH. Primer sequences were designed and synthesized as shown in Suppl. Table 2.

### Orthotopic mouse models of liver cancer and treatment

Orthotopic mouse models of liver cancer were established using HepG2-GFP liver cancer cells as previously described [20, 38, 39, 50]. The tumor-bearing mice were randomized into 4 groups (7 animals/group) and treated with 10% 2-hydroxypropyl-β-cyclodextrin (HPBCD) (Sigma), MLN4924 (30 mg/kg, s.c.), CQ (Sigma) (50 mg/kg, i.p.) or MLN4924 in combination with CQ (MLN4924+CQ) twice a day, on a 3-days-on/ 2-days-off schedule for 11 cycles within 56 days. Tumor size was measured with an Olympus OV100 imaging system twice a week as described [20, 38, 39]. Tumor tissues were harvested, photographed and weighed. Animal studies were performed in accordance with animal protocol procedures approved by the Institutional Animal Care and Use Committee of Fudan University.

### Immunohistochemistry

Paraffin-embedded tumor tissues were sectioned to 4-μm thickness and mounted on positively-charged microscope slides. Citric acid (Sigma) (pH 3.0) was used for antigen retrieval. Immunohistochemistry was performed by a GTVisionTMIII Detection System/Mo&Rb (Gene Tech Company Limited, Shanghai, China) using NOXA or cleaved caspase 3 antibody.

### Statistical analysis

The statistical significance of differences between groups was assessed using the GraphPad Prism5 software. The unpaired 2-tailed *t* test was used for the comparison of parameters between groups. The level of significance was set at *P* < 0.05.

## SUPPLEMENTARY FIGURES AND TABLES


