# Supplementary material for: Synergistic inhibition of autophagy and neddylation pathways as a novel therapeutic approach for targeting liver cancer
Source: Oncotarget. 2015 Mar 13;6(11):9002–17. doi: 10.18632/oncotarget.3282 (PMC4496198; doi:10.18632/oncotarget.3282)
Supplement: Supplementary file 1 [file oncotarget-06-9002-s001.pdf]

SUPPLEMENTARY FIGURES AND TABLES

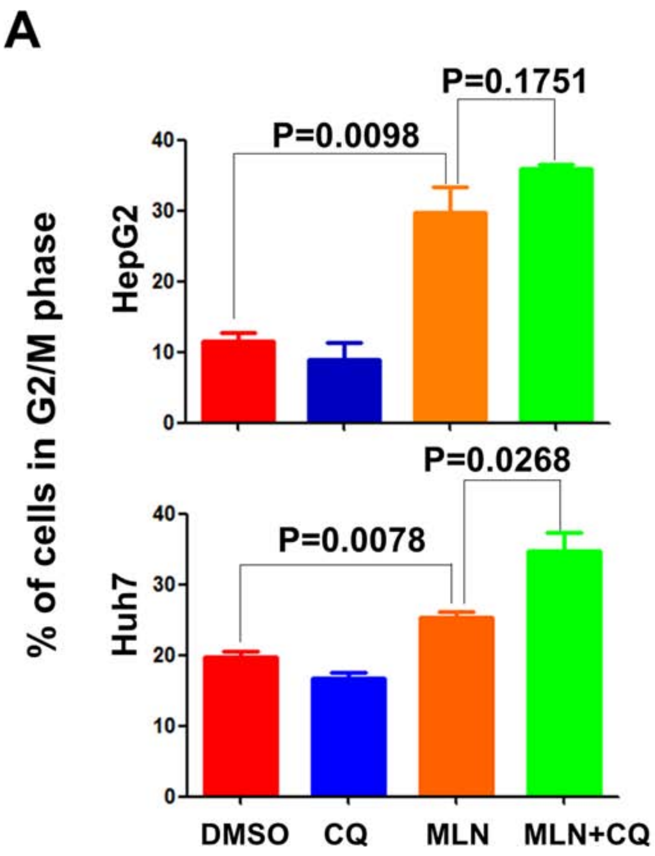

Supplementary Figure S1: The effect of autophagy inhibition on cell cycle. (A) Cell cycle arrest is triggered by MLN+CQ or MLN4924 treatment alone. HepG2 and Huh7 cells were treated with CQ (10 µM), MLN4924 (0.33 µM) or both for 24 hours and the cell cycle profile of treated cells is determined by flow cytometry, the distribution of G2/M phase is showed. **(B)** The effect of autophagy inhibition on the expression of cell cycle related proteins. Cells were treated as described in (A). Cell extracts were prepared, and equal amounts of protein were separated by SDS-PAGE and subjected to immunoblotting analysis with the indicated antibodies. Tubulin served as a loading control.

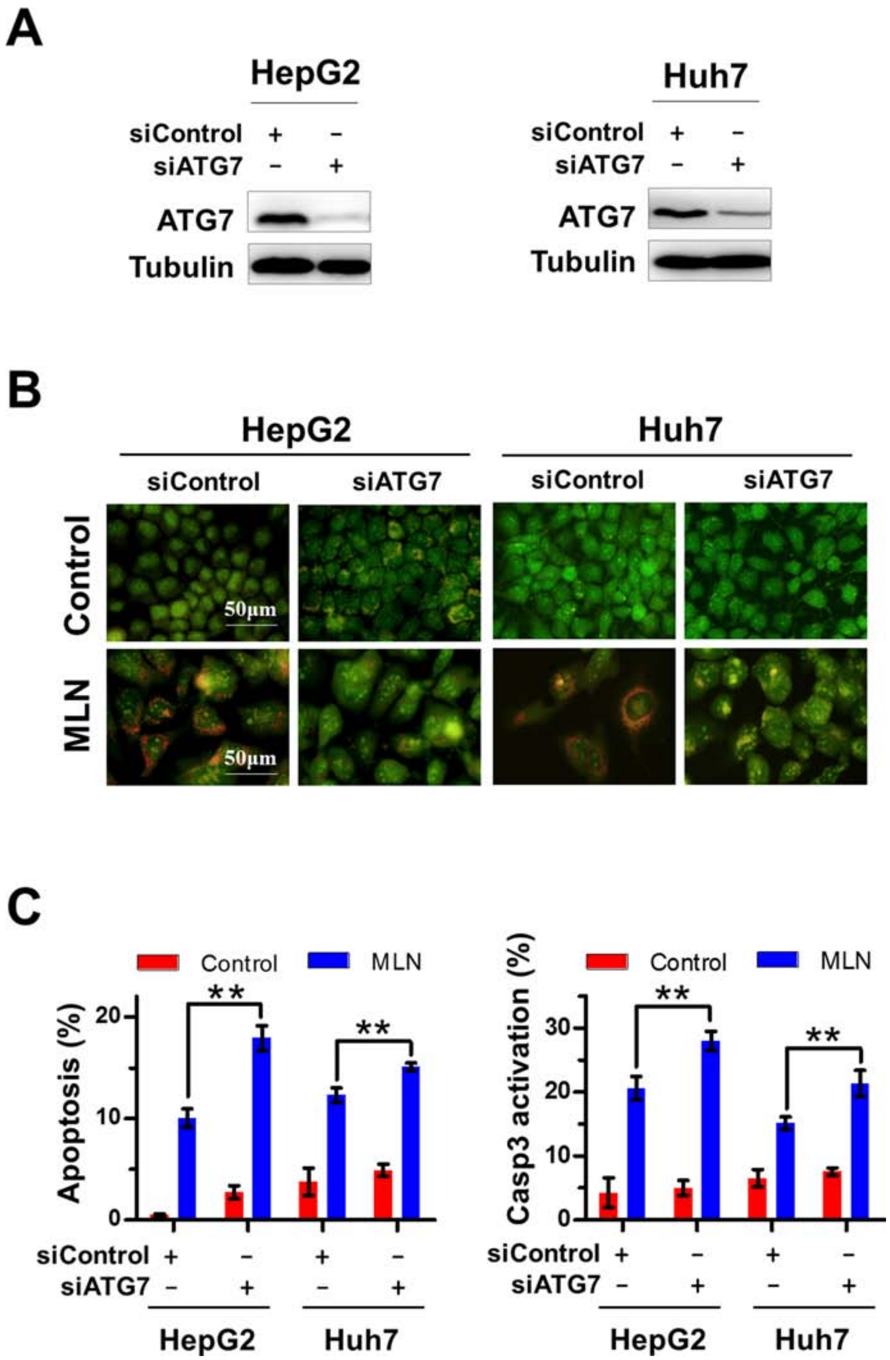

Supplementary Figure S2: Knockdown of ATG7 increases MLN4924-induced apoptosis. Cells transiently transfected with control siRNA or ATG7 siRNA for 24 hours and then were treated with MLN4924 (0.33 μM) for 72 hours. Knockdown efficiency was determined by immunoblotting **(A);** blockage of autophagy was determined by AO staining **(B)**; Apoptotic cell death was analyzed by the Annexin V-FITC/PI and caspase-3 activity assays **(C)**. All data are representative of three independent experiments (***P* < 0.01, *n* = 3).

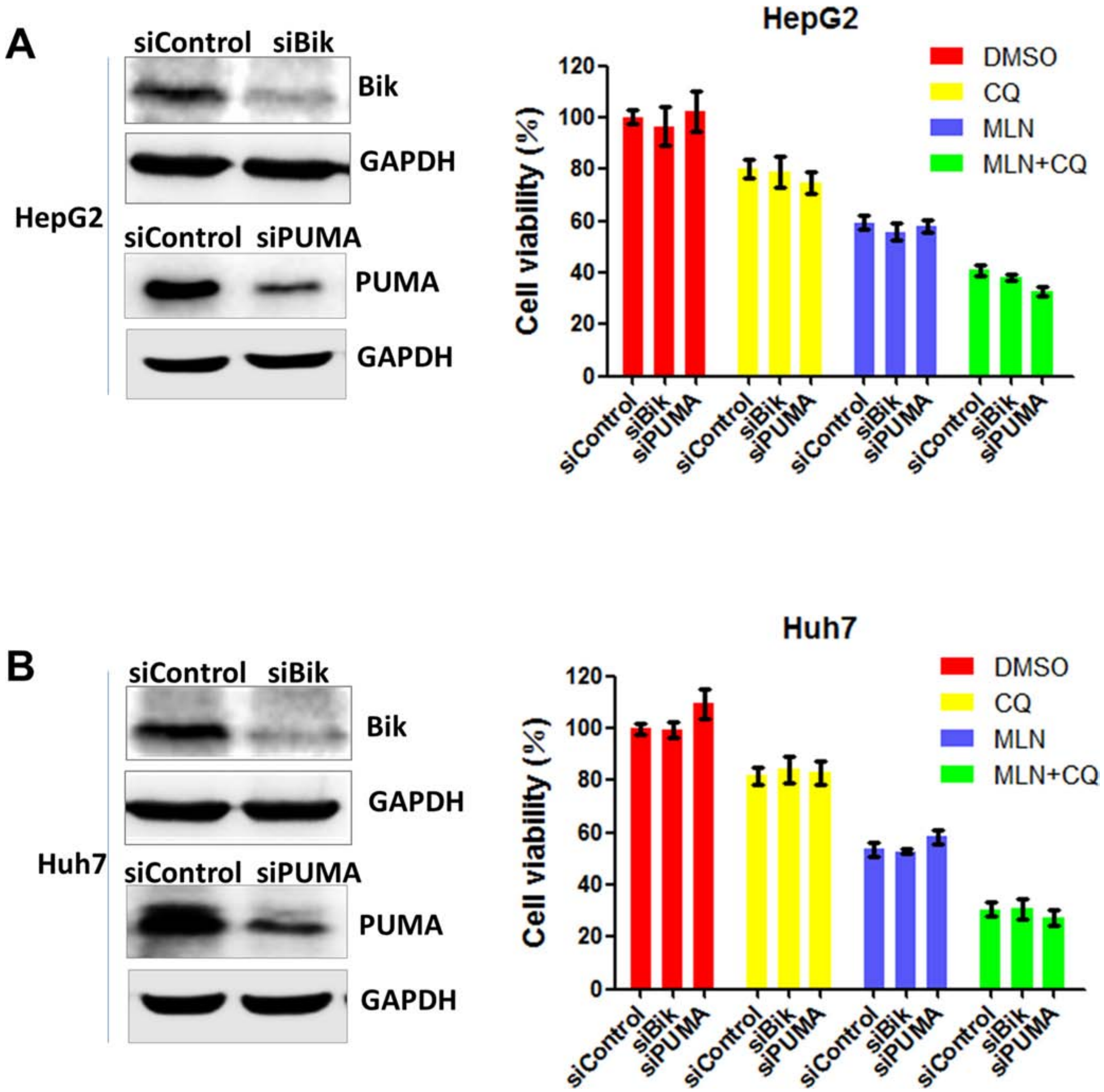

Supplementary Figure S3: Effect of down-regulation of BIK or PUMA on liver-cancer-cell proliferation inhibition induced by MLN4924+CQ. Cells transiently transfected with control siRNA, Bik or Puma siRNA for 24 hours and then were treated with MLN4924 (0.33 µM) for 72 hours. Knockdown efficiency was determined by immunoblotting **(A);** Cell viability was measured using the ATPLite assay **(B)**.

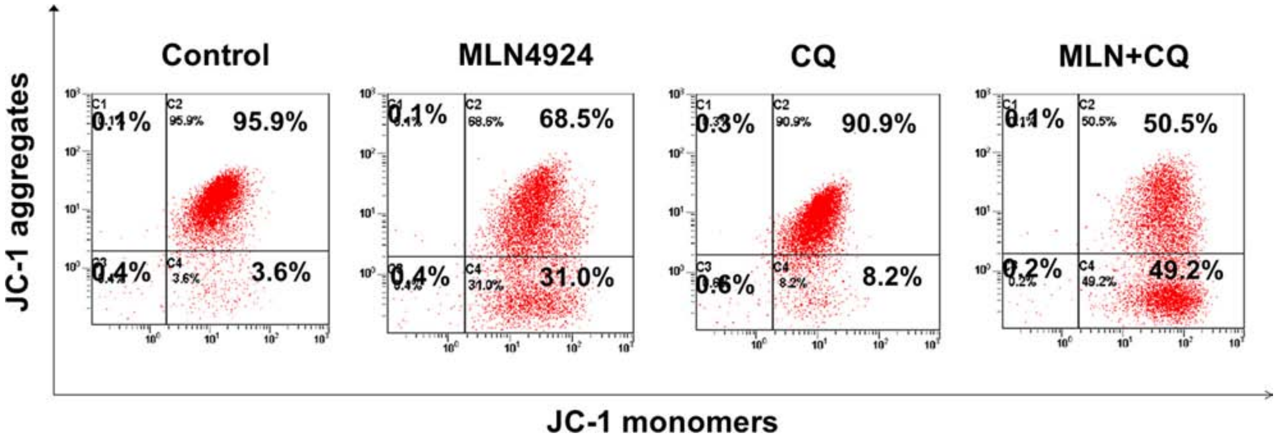

Supplementary Figure S4: Blockage of autophagy enhances mitochondrial membrane depolarization. HepG2 cells were treated with drugs as indicated. Mitochondrial membrane depolarization was detected with mitochondrial membrane potential assay kit with JC-1, according to the manufacturer's protocol (Yeasen, shanghai, China). All data are representative of three independent experiments.

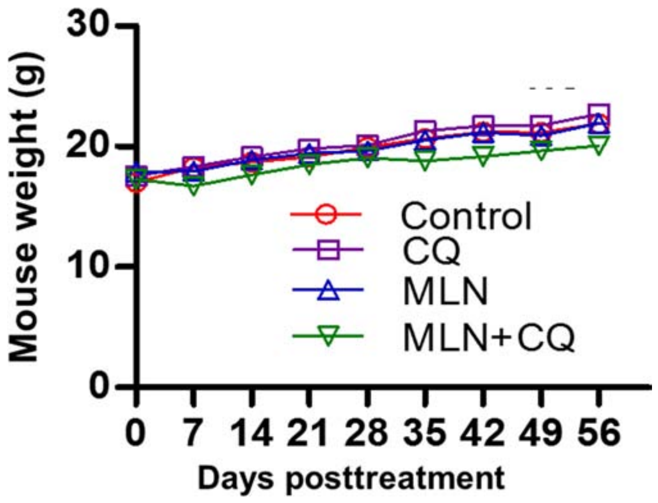

Supplementary Figure S5: No obvious toxicity against body weight gain was observed during treatment. Body weight of the animals was measured twice a week during the treatment period.

Supplementary Table S1. siRNA sequences used to silence target genes

Targeted Gene	Sequence
ATG7	5′-GGAGUCACAGCUCUUCCUU-3′
NOXA-1	5′-GUAAUUAUUGACACAUUUC-3′
NOXA-2	5′-GGUGCACGUUUCAUCAAUUUG-3′
Bik-1	5′-CGGUUUCACCACACUUAAG-3′
Bik-2	5′-ACGACCAGACUGAGGACAU-3′
Bik-3	5′-GCAGUGACGCAUUGGCCCU-3′
Puma-1	5′-GGGUCCUGUACAAUCUCAU-3′
Puma-2	5′-UCUCUGCACCAUGUAGCAU-3′
Puma-3	5′-CGAGAUGGAGCCCAAUUAG-3′
CDT1-1	5′-CGUGGAUGAAGUACCCGACUU-3′
CDT1-2	5′-GCAAUGUUGGCCAGAUCAAUU-3′
ORC1-1	5′-CUGCACUACCAAACCUAUA-3′
ORC1-2	5′-AAGGUCCCGGCUCAAGCAUGU-3′
Control	5′-UUCUCCGAACGUGUCACGU-3′

Supplementary Table S2. Primers used to detect the mRNA level of *NOXA*, *Bik* and *Puma* with Q-PCR

Gene	Sequence
NOXA-forward	5′-GGAGATGCCTGGGAAGA-3′
NOXA-reverse	5′-TTCTGCCGGAAGTTCAGT-3′
Bik-forward	5′-TTCATCTACGACCAGACT-3′
Bik-reverse	5′-ATCTCCAGAACCTCATTATG-3′
Puma-forward	5′-GCCCAGCCTGTAAGATAC-3′
Puma-reverse	5′-TTCCACTGTTCCAATCTGA-3′
GAPDH-forward	5′-AAAGGGTCATCATCTCTG-3′
GAPDH-reverse	5′-GCTGTTGTCATACTTCTC-3′

Supplementary Table S3. Analysis of the expression of apoptosis-regulatory genes by human apoptosis PCR array

position	Gene	MLN/ DMSO	CQ/ DMSO	MLN-CQ/ DMSO	MLN-CQ/ MLN
A1	DFFA	1.91	–1.27	1.57	–1.21
A2	CASP9	2.17	–1.01	1.83	–1.19
A3	CIDEA	1.39	1.06	12.87	9.26
A4	CD27	2.94	–1.90	12.15	4.13
A5	TNF	–8.61	1.99	–9.83	–1.14
A6	CIDEB	–1.63	1.34	–2.27	–1.39
A7	TNFRSF10B	2.28	2.29	3.39	1.49
A8	TNFRSF10A	3.63	5.28	14.25	3.92
A9	TP53	1.70	1.28	1.24	–1.37
A10	CD70	5.97	–1.13	19.33	3.24
A11	BCL2	–21.42	–70.33	–10.08	2.13
A12	BCL2L1	4.02	2.14	3.60	–1.12
B1	DAPK1	1.00	1.55	1.52	1.51
B2	NFKB1	1.65	1.45	2.69	1.63
B3	CASP6	–1.90	–1.01	–3.16	–1.67
B4	IGF1R	-7.61	–17.22	–18.85	–2.48
B5	AKT1	–3.24	–8.04	–5.16	–1.60
B6	GADD45A	4.31	2.07	15.92	3.69
B7	XIAP	2.92	1.60	5.20	1.78
B8	AIFM1	–1.23	–1.02	–1.76	–1.43
B9	CASP3	1.84	1.37	1.98	1.08
B10	CD40LG	–8.23	–21.25	–16.11	–1.96
B11	FADD	–1.10	1.03	2.21	2.43
B12	APAF1	3.06	2.47	3.98	1.30
C1	CASP7	2.16	1.87	2.42	1.12
C2	BIRC3	132.62	10.95	430.37	3.25
C3	BIRC2	2.48	1.65	3.76	1.51
C4	RIPK2	1.14	1.34	1.21	1.06
C5	CASP4	–1.33	2.12	1.13	1.50
C6	CASP5	–1.21	1.84	–1.07	1.13
C7	CASP1	–1.37	2.72	2.50	3.41
C8	CASP2	2.15	1.40	2.77	1.29
C9	BIRC5	–1.34	–1.51	1.16	1.56
C10	CASP10	2.62	1.54	1.04	–2.52

(*Continued*)

position	Gene	MLN/ DMSO	CQ/ DMSO	MLN-CQ/ DMSO	MLN-CQ/ MLN
C11	CASP8	4.35	2.26	2.80	–1.55
C12	FASLG	9.37	4.80	22.00	2.35
D1	IL10	–22.64	–27.02	–10.83	2.09
D2	TP73	–3.54	2.83	–14.46	–4.09
D3	CD40	1.78	–1.46	6.99	3.92
D4	NOD1	–2.02	–1.03	–3.92	–1.94
D5	BRAF	–1.45	1.18	–1.03	1.40
D6	MCL1	2.87	1.67	4.07	1.42
D7	ABL1	14.87	4.66	14.72	–1.01
D8	BAG1	–1.68	–1.19	–1.45	1.16
D9	BAG3	2.20	1.09	1.81	–1.21
D10	BCL10	–1.32	–1.28	1.41	1.86
D11	BCL2A1	3.63	1.40	14.25	3.92
D12	BCL2L10	3.63	1.40	14.25	3.92
E1	BCL2L2	3.30	1.95	4.24	1.28
E2	BFAR	1.31	1.33	2.07	1.58
E3	BIRC6	1.28	1.22	1.04	–1.23
E4	BNIP2	1.52	1.15	2.64	1.74
E5	BNIP3	–4.49	1.72	–3.92	1.14
E6	BNIP3L	1.79	2.03	2.66	1.49
E7	CASP14	1.36	–1.05	12.52	9.22
E8	CFLAR	1.30	–1.43	1.69	1.30
E9	CRADD	–1.63	1.50	–2.14	–1.31
E10	CYCS	–1.15	–1.78	–1.35	–1.17
E11	DIABLO	–1.03	–1.41	–1.69	–1.65
E12	FAS	–1.49	–1.18	2.52	3.77
F1	STAT3	6.82	1.61	8.90	1.31
F2	LTA	6.89	1.04	10.53	1.53
F3	LTBR	1.71	1.32	2.06	1.20
F4	NAIP	1.70	1.89	1.27	–1.34
F5	NOL3	1.30	1.28	2.17	1.67
F6	PYCARD	5.25	3.44	17.10	3.26
F7	TNFRSF11B	4.76	2.09	4.81	1.01
F8	TNFRSF1A	2.43	2.06	1.92	–1.27
F9	TNFRSF1B	1.91	1.44	1.84	–1.04
F10	TNFRSF21	5.85	1.19	3.67	–1.59

(*Continued*)

position	Gene	MLN/ DMSO	CQ/ DMSO	MLN-CQ/ DMSO	MLN-CQ/ MLN
F11	TNFRSF25	–1.83	2.10	1.37	2.51
F12	TNFRSF9	68.01	19.97	210.27	3.09
G1	TNFSF10	–1.47	1.92	–1.51	–1.03
G2	TNFSF8	34.24	1.40	14.25	–2.40
G3	TP53BP2	1.96	2.86	2.76	1.40
G4	TRADD	13.50	2.32	16.03	1.19
G5	TRAF2	2.20	1.31	2.02	–1.09
G6	TRAF3	1.58	1.55	1.56	–1.02
G7	STAT1	1.80	–1.36	1.87	1.04
G8	CDC2	2.02	1.43	2.08	1.03
G9	BAD	31.58	17.71	102.26	3.24
G10	BCL2L11	2.72	–1.37	4.78	1.76
G11	BID	1.63	–1.20	1.50	–1.09
G12	**Bik**	35.86	1.45	38.66	1.08
H1	BAX	1.81	1.56	2.93	1.62
H2	**NOXA**	5.21	7.08	60.83	11.67
H3	**Puma**	2.58	4.42	14.30	5.55
H4	BIRC7	–1.02	–2.02	4.51	4.62
